# Complete Genome Sequence of “*Candidatus* Phytoplasma asteris” QS2022, a Plant Pathogen Associated with Lettuce Chlorotic Leaf Rot Disease in China

**DOI:** 10.1128/mra.00306-23

**Published:** 2023-05-25

**Authors:** Xiao-Hua Yan, Jixiu Lin, Yanzhi Liu, Peizhi Huang, Jianmi Liu, Qibin Hu, Yalu Li, Shen-Chian Pei, Weijie Huang, Chih-Horng Kuo

**Affiliations:** a Institute of Plant and Microbial Biology, Academia Sinica, Taipei, Taiwan; b Yongan Crop Protection and Quarantine Station, Yongan, Fujian, China; c Shanghai Center for Plant Stress Biology, CAS Center for Excellence in Molecular Plant Sciences, Chinese Academy of Sciences, Shanghai, China; d Sanming Crop Protection and Quarantine Station, Sanming, Fujian, China; Wellesley College

## Abstract

The complete genome sequence of “*Candidatus* Phytoplasma asteris” QS2022, which consists of one 834,303-bp circular chromosome, is presented in this work. This bacterium is associated with lettuce chlorotic leaf rot disease in Fujian Province, China.

## ANNOUNCEMENT

Lettuce chlorotic leaf rot disease (LCLRD) is an emerging disease in China. It was first reported in Yong’an County, Fujian Province, in 2005. Since then, it has posed a persistent threat to the local lettuce production industry, causing significant economic losses every year. The causative agent, a 16SrI-B subgroup phytoplasma, is transmitted by the leafhopper Macrosteles striifrons and specifically invades the plant phloem (1). After infection, apical leaves become elongated and pale, growing buds rot, and plants eventually die. Because phytoplasmas are uncultivated, we conducted shotgun sequencing of an infected plant to study the genome of this bacterium. All kits were used according to the manufacturer’s protocols, and all bioinformatics tools were used with the default settings unless stated otherwise.

A symptomatic lettuce plant was collected in Qingshui Village of Yong’an County in October 2022. Pale apical leaves and buds were used for DNA extraction with the Hi-DNAsecure plant kit (DP350; Tiangen Biotech). For Illumina sequencing, the library was prepared using the VAHTS Universal Plus DNA library prep kit (ND617-C3-02; Vazyme), followed by sequencing on the Illumina NovaSeq 6000 platform to generate 9.1 Gb of 150-bp paired-end reads. For Oxford Nanopore Technologies (ONT) sequencing, DNA fragments of >10 kb were collected using the BluePippin system (Sage Science), followed by processing with NEBNext formalin-fixed, paraffin-embedded (FFPE) DNA repair mix and the NEBNext Ultra II end repair/deoxyribosyladenine (dA)-tailing module (New England Biolabs). The library was prepared using the ONT ligation sequencing kit (SQK-LSK109) without shearing and sequenced using PromethION flow cells (FLO-PRO002) on PromethION48. Guppy v3.2.6 was used for base calling and adapter trimming, which produced 618,488 reads totaling 9.9 Gb and with an *N*_50_ of 25.4 kb.

All Illumina and ONT reads were mapped to complete genome assemblies of 16SrI-B phytoplasmas (accession numbers GCF_000009845, GCF_001712875, and GCF_004214875) to identify bacterial reads using BWA v0.7.17 (2) and Minimap2 v2.15 (3), respectively. “*Candidatus* Phytoplasma asteris” OY-M (4) was found to have ~99% genome-wide average nucleotide identity with this new strain, QS2022, and was used to extract bacterial reads. The Illumina reads with an alignment score above 30 were extracted, trimmed using a Q20 cutoff, and combined with the ONT reads with an alignment score above 1,000 for assembly based on Trycycler v0.5.3 (5). The procedure of gene prediction and annotation was based on that described in our previous work (6, 7). For gene prediction, RNAmmer v1.2 (8), tRNAscan-SE v1.3.1 (9), and Prodigal v2.6.3 (10) were used. The annotation was based on the homologs in other phytoplasmas as identified by OrthoMCL v1.3 (11), followed by manual curation based on BlastKOALA v.12 (12) and GenBank (13). Putative secreted proteins were identified using SignalP v5.0 (14) and filtered by TMHMM v2.0 (15).

The assembly produced a single 834,303-bp circular contig with 27.6% G+C content, which represents the chromosome; no plasmid was found. The Illumina and ONT reads provided 547- and 136-fold coverage, respectively. The contig was rotated to have *dnaA* as the first gene. The annotation contains 6 rRNA genes, 32 tRNA genes, 819 protein-coding genes, and 36 pseudogenes.

### Data availability.

This whole-genome shotgun project has been deposited in NCBI under the accession number PRJNA937408 (https://www.ncbi.nlm.nih.gov/bioproject/PRJNA937408). The raw reads have been deposited at the NCBI Sequence Read Archive under the accession numbers SRX19478431 (https://www.ncbi.nlm.nih.gov/sra/SRX19478431) and SRX19478432 (https://www.ncbi.nlm.nih.gov/sra/SRX19478432). The genome sequence has been deposited at GenBank under the accession number CP120448 (https://www.ncbi.nlm.nih.gov/nuccore/CP120448).
